# Does the early phase of aging affect the morphology of biceps brachii and torque and total work of elbow flexors in healthy volunteers?

**DOI:** 10.1590/1414-431X2023e12202

**Published:** 2023-02-10

**Authors:** A.C. Mattiello-Sverzut, E.J. Martins

**Affiliations:** 1 Departamento de Ciências da Saúde, Faculdade de Medicina de Ribeirão Preto, Universidade de São Paulo, Ribeirão Preto, SP, Brasil

**Keywords:** Arm, Biopsy, Humans, Muscle strength dynamometer, Torque

## Abstract

Upper and lower limbs can be affected by several diseases and changes related to current life habits, such as the sedentarism, technological advances, and even eating habits. This cross-sectional study investigated morphological adaptations of the biceps brachii muscle and the performance of the elbow flexors in healthy individuals in the early phase of aging. Thirty-two volunteers were separated according to age range (3rd, 4th, and 5th decades of life) and sex. Smaller diameters and subtypes of fibers were evaluated using muscle biopsies, and peak torque and total work were assessed using an isokinetic dynamometer. The variables were compared considering sex and decade, using mixed-effects linear models. The smaller diameter of all fiber types did not differ significantly between age groups for either sex. The proportion of oxidative fibers was reduced in male participants in the 4th (-20%) and 5th (-6%) decades of life compared to the 3rd decade, and there was an increase in the number of oxidative fibers in women from the 4th (+14%) to the 5th decade of life. There were no significant changes in the peak torque and total work between the analyzed age groups. The early phase of aging starts with alterations in the proportion of fibers, with a decrease in oxidative fibers in men and an increase in oxidative fibers in women. Smaller diameter, torque, and total work did not change over these decades of life.

## Introduction

The performance of many activities of daily living (ADL) requires quality of movement, and this in turn is a combination of motor skills and active range of motion, and muscle strength. Upper and lower limbs can be affected by aging (1) and several diseases, as well as changes related to life habits, such as sedentarism, technological advances, and even eating habits. Because of the strong impact of lower limb muscle function on falls and fall-related injuries in older adults, most of the scientific literature focuses on the cellular and functional changes associated with lower limb muscles. In contrast, the involvement of upper limbs during the aging process is scarcely mentioned.

The upper limbs are responsible for an extensive repertoire of movements and perform most vital tasks such as drinking, eating, dressing, or personal care. Oosterwijk et al. (2) compiled and synthesized data from the literature showing the ADL tasks performed with shoulder and elbow in healthy participants. The authors pointed out that the performance of many ADL tasks required a high degree of elbow flexion (from 130° up to 150°). Bazzucchi et al. (3) observed that older men showed longer isometric endurance time and less absolute torque measured at different angular velocities (15, 30, 60, 90, 120, and 150°s^-1^). Regarding the elbow extension torque, a cross-sectional study reported decreases of 20 to 40% and a longitudinal study demonstrated decreases of 1 to 5% in elderly participants compared to young participants (4).

Recently, Larsson et al. (5) presented an excellent review of aging-related changes in skeletal muscle tissue. As was reported four decades ago, human muscle fibers expressing fast myosin isoforms had a more intense reduction in cross-sectional areas than slow fibers (6). This was due to the increase in the proportion of type 1 fibers (due to the conversion of type 2 fibers into type 1 fibers), although the total cross-sectional area did not change significantly (7). Additionally, the central nervous system, peripheral nerves, motor units, neuromuscular junctions, muscle tissue, and mechanical properties of tendons participate in a process that involves not only muscle atrophy and loss of strength but also slowing of movements and postural adjustments (5). However, the study of age-related changes of upper limb muscles, which basically work in an open kinetic chain, is rarer. This means that the scientific findings for the lower limbs cannot be extrapolated to the upper limbs. Studies have shown aging effects on the biceps brachii, such as a reduction in the size of type 1 (8) and type 2 fibers (8,9) in young and old male individuals. Inversely, Klitgaard et al. (10) did not identify a significant variation in the cross-sectional area of types 1, 2A, and 2B fibers of this muscle when comparing four healthy young men (mean age of 28 years) with four older adult men (mean age of 69 years). In later aging, there is minimal loss of muscle mass and relevant reduction in muscle function (11).

For the clinical care of patients with upper limb disabilities, traumatic lesions in athletes, or for any clinical trial examining a new drug treatment or surgery, quantitative assessment measures are needed to precisely track the progress of muscle performance and morphology. For example, the cross-sectional area and fiber type distribution of biceps brachii may be extensively used to assist in diagnostic reports of neuromuscular diseases and other types of clinical research. In addition, muscle strength and performance can be evaluated using the gold standard equipment, the isokinetic dynamometer (12,13). Orthopedic and rheumatology specialists in particular use this device to identify the efficacy of surgery, training, and prevention of muscle or articular lesions (14-16).

The challenge remains in detecting minor changes of the upper limbs associated with aging, diseases, and disabilities. Therefore, investigating the aging effects on anatomical (different fiber types and proportions), dynamic (using a gold standard isokinetic protocol), and functional measurements (standardized clinical scales), considering sex and age, would improve the understanding of human reference values for minimally required tasks related with ADL.

Therefore, the purpose of this study was to examine the adaptations of the elbow flexor muscles considering the morphological approach and muscle performance evaluation using the isokinetic test at different velocities of movement in healthy individuals of both sexes in the early phase of aging: the 3rd, 4th, and 5th decades of life. We hypothesize that if muscle mass changes minimally in later aging, it is likely that in the early phase of aging, when the movement repertoire required for ADL does not change, the greatest adaptations in upper limbs are morphological rather than in the muscle performance.

## Material and Methods

### Participants

This cross-sectional study included 32 healthy volunteers (convenience sample), divided into groups according to age range and sex: 3rd decade (n=12; 7 females); 4th decade (n=10; 5 females); and 5th decade (n=10; 5 females). The inclusion criteria were: age between 20 and 50 years, both sexes, recreational physical activity level, no immobility in the last 7 days, and presence of morphologically normal muscle samples (few artifacts or mild fiber size variability). The exclusion criteria were athletes (who competed in one or more sports), volunteers with a family history of myopathy, uncontrolled or treated hypertension or hypotension, history of fracture or recent surgery in the preferred upper limb (less than one year), history of reactions to xylocaine (or other local anesthetics), and systemic diseases.

The volunteers were asked about their physical activity level and health status using a simple questionnaire following the recommendations of the WHO Guidelines on Physical Activity and Sedentary Behavior in order to identify the amount of regular aerobic and/or muscle-strengthening exercise per day and per week (17). All volunteers signed a consent form. The Research Ethics Committee of the Ribeirão Preto Medical School, University of São Paulo, Brazil, approved this study (Protocol number 6068/2006).

### Muscle biopsy and morphometric analysis

Of the 32 volunteers who participated in this study, six samples were found to have freezing artifacts that prevented morphometric analysis from being performed. Of the 26 remaining volunteers, 14 were male and 12 were female (mean age=34.1; SD=8 years). All volunteers had previous experience with local anesthesia, i.e. for dental treatments, and showed no reaction to the anesthetic. The procedure for obtaining the muscle fragment was performed on the non-preferential upper limb and consisted of a percutaneous needle biopsy. Before the biopsy, the region was cleansed and anesthetized using 2% lidocaine without epinephrine. A #11 scalpel blade was then used to make a small incision of 0.5 cm in the skin, subcutaneous tissue, and muscle fascia. A 4.5-mm biopsy needle with suction (Bergström model) was then inserted (18). The fragment from the biceps brachii muscle was obtained from the medium-distal portion between an imaginary line from the tuberculum majus humeri to the elbow crease. Immediately after extraction, all fragments were covered with talc, frozen in liquid nitrogen, and stored in a cryotube at -80°C. Afterwards, serial cross-sections (5 µm) were made using a Leica CM 1850 UV microtome (−25°C) (Leica Instruments GmbH, Germany).

The biceps brachii muscle fragments were stained with hematoxylin and eosin (HE) for the general morphological evaluation and processed for detection of mATPase (E.C.3.6.1.3.) activity by pre-incubation in acid and alkaline media in order to identify the different fiber types and subtypes (19). A neuropathologist performed the analysis of the slides to confirm the absence of morphological signs indicative of neuromuscular diseases. No volunteer was excluded for this reason, but the slides from six volunteers were excluded from the morphometric analysis because they presented freezing artifacts. The images were acquired with a Leica DC 300FX camera coupled to a Leica DM 2500 binocular microscope and connected to an IBM personal computer (USA). The classification of fiber types was based on the stability of mATPase after pre-incubation in alkaline and acid media (19). The morphometric analysis used the interactive mode of the LAS software (Leica), and in each muscle sample, at least 100 of all fiber types were measured to obtain the smallest diameter (9). To determine fiber distribution, images of three random fields (40× objective) per volunteer were collected from the fragments processed for myofibrillar ATPase. The fiber types of the biceps brachii muscle were classified using slides incubated at pH 9.8, 4.6, and 4.3. It was possible to determine pure fiber types (1, 2A, and 2D) and intermediate/hybrid fiber types (2C and 2AD). Type 1 fibers were classified using pH 9.8; types 2A, 2D, and 2AD fiber were classified using pH 4.6. The pH value of 4.3 was used to confirm the classification of type 2C fiber.

### Isokinetic evaluation

The isokinetic evaluation was performed using an isokinetic dynamometer (Biodex Multi Joint System 2^®^, Biodex Medical Systems, Inc., USA) from the Department of Physiotherapy, Federal University of São Carlos, Brazil.

First, upper limb muscles were “warmed up” using an unloaded cycle ergometer for 3-min. Afterwards, the volunteers were seated in the isokinetic dynamometer chair with a back angle of 90° and stabilized in the chair with belts placed over the chest, pelvis, and tested arm (preferred side). The mechanical axis of rotation of the dynamometer was aligned with the lateral epicondyle of the humerus. The shoulder was positioned at 30° in the plane of the scapula, 30° abduction in the frontal plane, 0° flexion, and the forearm in supination. The device for fixation of the forearm was placed in the middle third of the forearm, and the application of resistance was placed in the middle third of the metacarpals so that the individual could hold the distal part of the resistance device.

Before recording data, two submaximal contractions were performed at each test velocity to familiarize the volunteer with the equipment. Then, the isokinetic evaluation was performed in the reciprocal, continuous, and concentric modes of elbow flexion and extension at five different angular velocities, using low velocities (60 and 90°s^-1^) to measure strength and moderate to fast velocities (180, 240, and 300°s^-1^) to evaluate power (12), chosen at random, with 15 repetitions each. Range of motion for elbow flexion was between 60 and 130°. A 10-min rest interval was established between isokinetic velocities in order to avoid the possible effects of muscle fatigue. All assessments were performed by the same evaluator. During the tests, standardized and constant verbal encouragement was provided to ensure that the volunteers exerted maximum strength during the contractions (20).

### Statistical analysis

Descriptive statistical analysis of the anthropometric data are reported as means (SD). For morphometric analysis, mean small fiber diameter and fiber distribution were compared considering sex and age group. For isokinetic analysis, the peak torque relative to body weight (PT - in percentage) and total work (TW, in Joules - J) of each volunteer were obtained at the five angular velocities. TW was obtained by the average of the performance in the 15 repetitions. The following comparisons were made: between age groups for the same sex, between sexes for the same age group, and between age groups for the same velocity and sex. Data were analyzed using linear mixed-effects models (21). Multiple comparisons were performed by contrasts. For each of the linear mixed-effects models, the normality of the residuals was checked using normal probability plots. All statistical analyses were performed using the software SAS^®^ 9.0 (USA), with the level of significance set at 0.05.

## Results

The general characteristics of the volunteers, according to each decade of life, are summarized in the Table 1.

**Table 1:** General characteristics of participants in each decade of life.

Variable	Muscle morphology			Isokinetic test		
	3rd decade	4th decade	5th decade	3rd decade	4th decade	5th decade
Volunteers (n)	9	8	9	12	10	10
Gender	5F/4M	3F/5M	4F/5M	7F/5M	5F/5M	5F/5M
Age (years)	24.1 (1.7)	34.4 (3.8)	44.2 (2.2)	34.1 (1.7)	44.4 (3.8)	54.2 (2.2)
Body mass (kg)	70.6 (13.4)	73.4 (13.8)	74 (22.5)	70.6 (13.4)	73.4 (13.8)	74 (22.5)
Height (cm)	170 (0.06)	171 (0.1)	170 (0.1)	170 (0.06)	171 (0.1)	170 (0.1)

Data are reported as mean (standard deviation). F: female; M: male.

### Fiber size and distribution

The first statistical approach explored if the mean small fiber diameter was significantly different between the sexes. Males presented higher values of all fiber types than females (P<0.05). Then, the consecutive statistical analysis separated the results of the volunteers according to sex. The comparison between the age groups showed no significant difference for all fiber types, for both males and females (data not shown). This means that the smaller diameter of type 1 fiber did not modify significantly from the 3rd to 5th decade of life for male and female volunteers. Table 2 shows the mean (95%CI) values of small diameter fibers obtained for all fiber types and both sexes, considering the interval from 20 to 50 years of age. It was observed that all fiber types of males showed higher values than of females, and the mean small fiber diameter of type 2A fibers was higher than the other fiber types, for both sexes.

**Table 2:** Small fiber diameter (in µm) considering the different fiber types and sexes.

Gender	Diameter				
	FT1	FT2A	FT2D	FT2AD	FT2C
Females	48.6*(48.0-49.1)	50.6*(49.5-51.8)	47.4*(44.9-49.8)	42.9*(42.0-43.7)	44.7*(43.2-46.2)
Males	53.7(53.1-54.3)	64.2(62.9-65.6)	52.0(48.6-55.4)	57.5(56.1-58.8)	54.6(52.6-56.6)

Data are reported as mean (95% confidence interval). FT1: type 1 fibers; FT2A: type 2A fibers; FT2D: type 2D fibers; FT2AD: type 2AD fibers FT2C: type 2C fibers. *P<0.05 compared to males (linear mixed-effects models).

**Figure 1:**
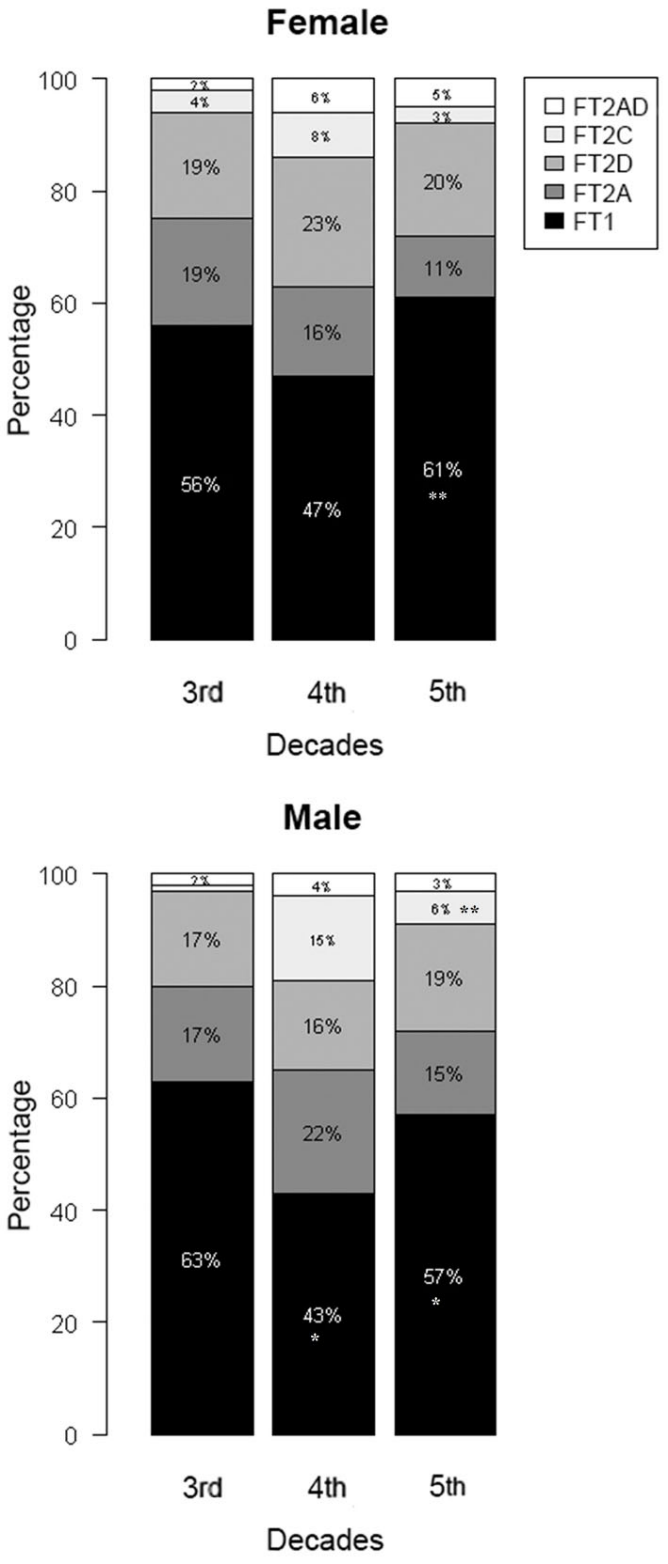
Percentage of fiber types of the biceps brachii in males and females in different decades of life. *P<0.05 compared to the 3rd decade; **P<0.05 compared to the 4th decade (linear mixed-effects models). FT1: type 1 fibers; FT2A: type 2A fibers; FT2D: type 2D fibers; FT2C: type 2C fibers; FT2AD: type 2AD fibers.

There was a significant decrease in the proportion of type 1 fibers of male participants in the 4th and 5th decades of life than of male participants in the 3rd decade of life (reduction of 20% in the 4th and reduction of 6% in the 5th decades; P<0.05) (Figure 1). We also observed a significant decrease in the proportion of type 2C fibers from the 4th to the 5th decade (reduction of 9% in the 5th decade; P<0.05). In females, we observed a significant increase in the proportion of type 1 fibers from the 4th to the 5th decade (increase of 14% in the 5th decade; P<0.05) (Figure 1).

### Isokinetic variables

**Figure 2:**
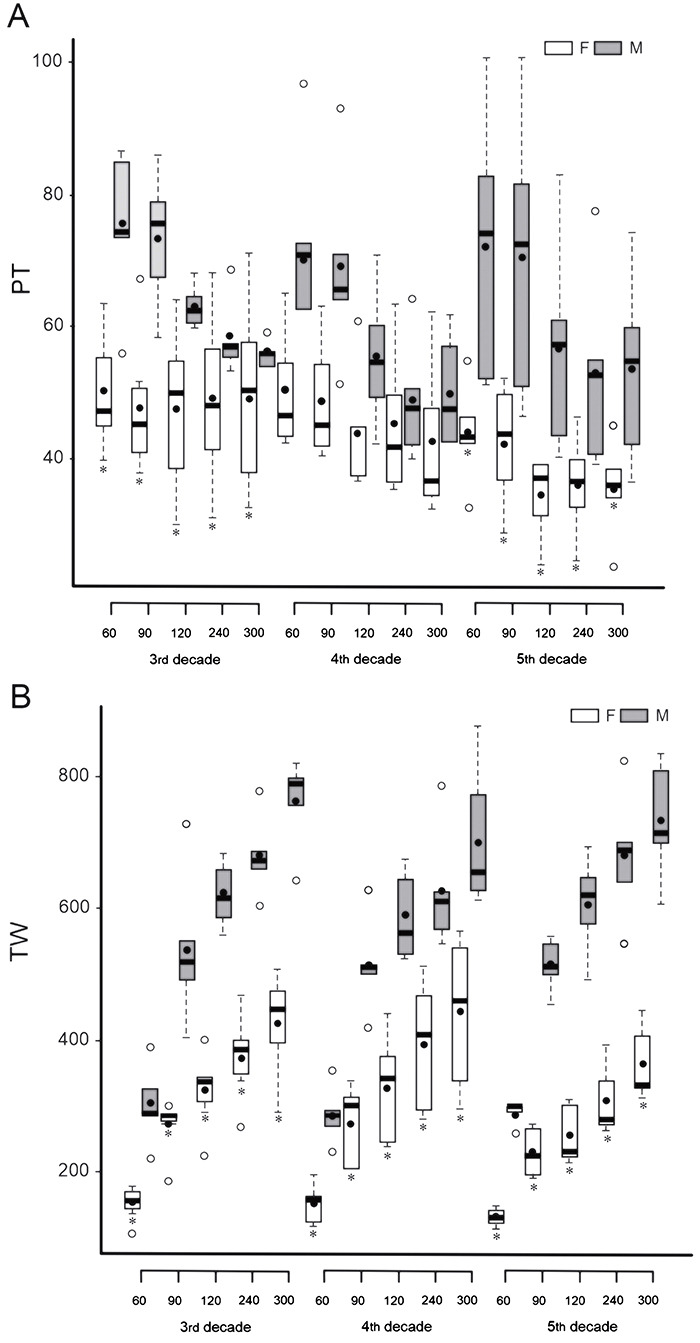
Peak torque (**A**) in Newton-meter relative to body weight and total work (**B**) in Joules obtained in the concentric isokinetic test of elbow flexion of volunteers in different decades of life at different angular velocities. PT: peak torque; TW: total work; F: females; M: males. Data are reported as median and interquartile ranges; °outliers. *P<0.05 compared to male group in the same decade of life and velocity (linear mixed-effects models).

The highest mean PT values were obtained by male volunteers in all decades and at all velocities; the highest PT value was from male volunteers in the 3rd decade, with a velocity of 60°s^-1^ (74.98±12.30%). The lowest PT value was from female volunteers in the 5th decade, with a velocity of 180°s^-1^ (34.32±6.79%). In the 3rd and 5th decades and at all velocities, the mean values of PT were significantly lower in female compared to male volunteers (P<0.05) (Figure 2).

The highest mean TW values were obtained by male volunteers in all decades and at all velocities, with the highest TW value being found for males in the 3rd decade, with a velocity of 300°s^-1^ (761.46±71.72 J). The lowest TW value was observed in females in the 5th decade, with a velocity of 60°s^-1^ (129.22±18.83 J). For the three decades analyzed and at all velocities, the mean values of TW were significantly lower in females compared to males (P<0.05) (Figure 2).

Mean PT and TW values of age groups of the same sex did not differ significantly (P>0.05).

PT and TW values obtained in the concentric isokinetic test of elbow flexion at different angular velocities are presented in Supplementary Table S1. Data from the comparison of the PT and TW between age groups for the same sex, between sexes for the age group, and between age groups for the same velocity and sex are presented in the Supplementary Tables S2, S3, and S4, respectively.

## Discussion

The present study showed that the smaller diameter of the different fiber types in the biceps brachii muscle did not differ significantly between decades, either for males or females. On the other hand, the proportion of oxidative fibers in males was lower in the 4th and 5th decades compared to the 3rd decade, and in females, there was a higher proportion of oxidative fibers in the 4th and 5th decades. Additionally, there were no changes in the performance of the elbow flexor muscle during the early phase of aging, since there were no significant changes in the PT and TW among the analyzed decades. Our hypothesis was confirmed since the morphological adjustments occurred before strength adjustments.

### Morphological differences

The size of muscle fibers changes and can convert from one type to another in response to stimuli, such as training or immobilization (22), and also with aging (5). However, in the present study, no change was observed in the small fibers of the biceps brachii muscle between the 3rd, 4th, and 5th decades for both sexes. Mattiello-Sverzut et al. (8) also did not observe variation in these muscle fibers with aging for females; however, in males, they identified a greater value for type 1 and type 2 small fibers between the 4th and 5th decades of life, and a quadratic pattern of regression of the diameter of these fibers from the 5th decade onward. Although these results seem contradictory, the quadratic model was influenced by data at the extremes of the age groups imputed in the regression referring to the 2nd, 6th, 7th, and 8th decades (8). Klein et al. (9) also identified atrophy of biceps brachii fibers, especially of type 2 fibers, when comparing male young adults (n=6; mean age 21) and male older adults (n=6; mean age 82). Therefore, the studies available in the literature do not contradict the present findings, since they used extreme age values.

In male volunteers, the proportion of type 1 fibers was lower in the 4th and 5th decades compared to the 3rd decade, and the proportion of type 2C fibers was lower in the 5th decade compared to the 4th. One hypothesis is that the lower number of type 1 and type 2C fibers in males is related to the continued conversion of these fiber types to type 2 fibers, a process that begins in childhood and progresses throughout maturity. Muscle fiber type distribution was investigated in human skeletal muscle in children, adolescents, and adults in the deltoid muscle (23) and vastus lateralis muscle (24), and it was observed that the type 1 fiber percentage is greater in male children, teenagers, and young adults than in older male adults. Likewise, type 2C fibers seem to actively participate in the conversion to type 2 fibers, as they are muscle fibers that are highly capable of undergoing transition to another type (25). Analyses of the biceps brachii muscle (superficial portion) from autopsies of adult male participants indicated a higher percentage of type 2 fibers (57.7%) than type 1 fibers (42.3%) (26). In addition, adult men have a proportionally smaller area of type 1 fibers because they have a larger area of type 2 fibers than women in various muscle groups (27). The results of the present study are in line with those mentioned above, as the volunteers showed higher mean values for small type 2A fibers compared to the other fiber types for both sexes and a significant difference between the sexes, with higher values for male volunteers.

In female volunteers, there was a significant increase in the proportion of type 1 fibers from the 4th to the 5th decade. Again, these findings can be related to the conversion of fiber types in aging muscles. The lower proportion of type 2 fibers and, secondly, the preferential atrophy of type 2 fibers leads to loss of muscle mass with advancing age and therefore a greater area with slow fibers in aging muscles (1). Furthermore, with age, there is a loss of alpha motoneurons via denervation, followed by some reinnervation of muscle fibers denervated by adjacent motor units, facilitating the conversion from one fiber type to another (20,25,28). Therefore, the increase in the proportion of type 1 fibers is an expected change during aging (1,22), and it seems to occur earlier in women (from the 4th decade of life) than in men, because during menopausal transition (from the pre-menopause to post-menopause) there is a decline in estradiol levels (29), which is the most important estrogen hormone. One of the functions of estradiol is to stimulate activation and, consequently, proliferation of satellite cells through specific estrogen receptors (ER-a and ER-b) (30), promoting muscle plasticity and regeneration (31), especially in type 1 fibers, which have greater blood capillary contribution (32).

### Muscle performance differences

The greatest age-related changes in strength occur in men (33), because the pattern of hormonal changes (testosterone, growth hormone, and IGF-1) is different between the sexes (4). However, in the present study, we observed lower values of PT (in the 3rd and 5th decades) and TW (in the 3rd, 4th, and 5th decades) of female elbow flexors. The decline in muscle strength in females begins during peri-menopausal phase and this phenomenon seems also to be partly estrogen-dependent. Randomized controlled clinical trials have indicated that hormone replacement therapy may prevent a decline in muscle performance (34). There are specific receptors for estradiol at the level of muscle fibers that respond to estrogenic hormonal control and stimulate satellite cell proliferation (35). In women, age-related reductions in estrogen levels can also affect muscle strength, because estrogen is converted to testosterone, which has an anabolic effect on muscle protein synthesis. Furthermore, both sex hormones can suppress inflammatory cytokines, such as interleukin (IL)-6, IL-1, and tumor necrosis factor-α (TNF-α), that exert catabolic effects on muscle (35).

### Interaction between muscle morphology and muscle performance

Part of the decrease in force or torque during aging is due to a decrease in the proportion of type 1 and type 2 fibers and preferential atrophy of type 2 fibers, causing loss of muscle mass (1,25). The maintenance of small fibers and the proportion of type 2A fibers in the biceps brachii of volunteers of both sexes over the three 10-year age groups analyzed contributed to the conservation of muscle mass and, consequently, of the PT and TW of the elbow flexors. Furthermore, it has been reported that the number of motor units in upper limb muscles is less altered than in lower limbs in humans, especially in proximal muscles, as biceps brachii, which are less susceptible to nerve supply deterioration (36), contributing to the conservation of muscle mass and upper limb functionality during aging.

The early age-related process of healthy men starts with the conversion of type 1 muscle fibers into type 2 fibers and the opposite occurs in women, similar to the alterations of older people over 60 years of age (20). The small fibers of the different fiber types and muscle performance, regardless of the velocity, do not change over these decades.

The limitations of the present study were the lack of the evaluation of the physical activity level, labor, or sporting activity of the volunteers using specific scales, and the absence of immunohistochemical analyses for MHC and PCR, which could complement the fiber conversion assessments.

To the best of our knowledge, this is an original study exploring morphological and muscle performance (using different test movement velocities) of elbow flexor muscles in healthy individuals in the early phase of aging. These data allow a more precise comparison across age groups and sexes providing quantitative assessment measures to follow the progress of muscle performance and muscle morphology in athletes and (or) in patients with upper limb disabilities. Further research is suggested to explore the molecular, biochemical, and histopathology of biceps brachii and PT and TW of elbow flexors in healthy volunteers for the age group above 50 years of age.

## Supplementary Material

Click here to view [pdf].
